# Supplementary material for: The Ability of a Novel Trypsin-like Peptidase Activity Assay Kit to Detect Red-Complex Species
Source: Diagnostics (Basel). 2022 Sep 8;12(9):2172. doi: 10.3390/diagnostics12092172 (PMC9497697; doi:10.3390/diagnostics12092172)
Supplement: Supplementary file 1 [file diagnostics-12-02172-s001.zip › diagnostics-1882883-supplementary.pdf]

Table S1 Culure methods of 40 oral bacteria using crossover examination for ADCHECK

	Bacterial Name	Strain No.	Culture medium	Growth condition	Culture time
1	*Bordetella pertussis*	NBRC107857	NBRC347	37℃	72h
2	*Candida albicans*	NBRC1385	NBRC108	24℃	24h
3	*Corynebacterium diphtheriae*	JCM1310	JCM28	37℃	48h
4	*Enterococcus durans*	NBRC100479	NBRC814	37℃	24h
5	*Enterococcus faecalis*	NBRC100480	JCM28	37℃	24h
6	*Haemophilus infuluenzae*	ATCC9006	ATCC814	37℃ 5% CO_2	48h
7	*Listeria monocytogenes*	JCM7671	JCM28	37℃ 5% CO_2	24h
8	*Moraxella catarrhalis*	ATCC8176	ATCC44	37℃ 5% CO_2	24h
9	*Mycoplasma orale*	NBRC14477	NBRC246	37℃ 5% CO_2	96h
10	*Mycoplasma pneumoniae*	NBRC14401	PPLO	37℃	137h
11	*Mycoplasma salivarium*	NBRC14478	NBRC823	37℃ 5% CO_2	141h
12	*Mycoplasma hominis*	NBRC14850	NBRC823	37℃ 5% CO_2	72h
13	*Proteus vulgaris*	NBRC3045	NBRC802	30℃	24h
14	*Pseudomonas aeruginosa*	NBRC12689	NBRC802	30℃	24h
15	*Serratia marcescens*	NBRC3046	NBRC802	30℃	24h
16	*Staphylococcus aureus*	NBRC102135	NBRC802	35℃	24h
17	*Staphylococcus epidermidis*	NBRC100911	NBRC802	37℃	24h
18	*Streptococcus agalactiae*	JCM5671	NBRC347	37℃	72h

19	*Streptococcus anginosus*	JCM12993	NBRC108	37℃	24h
20	*Streptococcus dysgalactiae subsp. dysgalactiae*	JCM5673	JCM28	37℃	48h
21	*Streptococcus mutans*	NBRC13955	JCM70	37℃	24h
22	*Streptococcus oralis*	JCM12997	JCM70	37℃	48h
23	*Streptococcus pneumoniae*	NBRC102642	JCM70	37℃	48h
24	*Streptococcus pyrogenes*	JCM5674	NBRC814	37℃ 5% CO_2	24h
25	*Streptococcus intermedius*	ATCC9895	JCM27	37℃ 5% CO_2	24h
26	*Streptococcus sanguis*	JCM5708	NBRC347	37℃	24h
27	*Neisseria gonorrhoeae*	ATCC19424	JCM70	37℃	48h
28	*Neisseria meningitidis*	ATCC13077	ATCC260	37℃	24h
29	*Neisseria sicca*	ATCC9913	JCM70	37℃	24h
30	*Neisseria subflava*	ATCC 19243	ATCC814	37℃ 5% CO_2	24h
31	*Streptococcus pyogenes*	ATCC12353(T12)	ATCC814	37℃ 5% CO_2	24h
32	*Streptococcus pyogenes*	ATCC 12962(T28)	ATCC44	37℃ 5% CO_2	48h
33	*Streptococcus pyogenes*	BAA-1066(M4)	ATCC44	37℃ 5% CO_2	24h

34	*Escherichia coli*	ATCC11775 (JCM1649)	ATCC260	37℃	24h
35	*Klebsiella pneumoniae*	ATCC13883 (JCM1662)	ATCC260	37℃	24h
36	*Citrobacter freundii*	JCM1657	ATCC260	37℃	24h
37	*Salmonella enteritidis*	IFO3313	ATCC260	37℃ 5% CO_2	24h
38	*Salmonella typhimurium*	IFO13245	ATCC3	37℃	24h
39	*Streptococcus dysgalactiae subsp. equisimilis*	ATCC12388	ATCC3	37℃	24h
40	*Streptococcus constellatus subsp. constellatus*	JCM12994	JCM75	37℃	24h
